# Safety of COVID-19 vaccines in pregnancy: a VAERS based analysis

**DOI:** 10.1007/s00228-023-03482-8

**Published:** 2023-03-24

**Authors:** Greta Santi Laurini, Nicola Montanaro, Domenico Motola

**Affiliations:** 1 Unit of Pharmacology, Department of Medical and Surgical Sciences, Alma Mater Studiorum, University of Bologna, Bologna, Italy; 2 Former Professor of Pharmacology at the Alma Mater Studiorum, University of Bologna, Bologna, Italy

**Keywords:** Adverse events, Vaccines, COVID-19, Safety, Pregnancy, Surveillance

## Abstract

**Purpose:**

Since vaccination against COVID-19 is recommended in pregnant people, we aimed to provide further evidence on the safety profile of COVID-19 vaccines in pregnancy.

**Methods:**

Data on COVID-19 vaccines adverse events following immunizations (AEFIs) in pregnant people were retrieved from the open-access Vaccine Adverse Event Reporting System (VAERS) from December 2020 to April 2022.

**Results:**

From December 2020 to April 1, 2022, a total of 4,869 reports involving pregnant women at COVID-19 vaccination were reported to VAERS. Among vaccines recipients, most belonged to the age group between 30 and 39 years old (3,029; 62.21%) and nearly half experienced an adverse event within 48 h of immunization (2,344; 48.14%). Overall, 21,816 suspected adverse reactions associated with COVID-19 vaccines were reported, and for as many as 80.43% of patients, they were described as non-serious. Most reactions occurred after administration of the mRNA-1273 (53.34%) and the BNT162b2 (40.68%) vaccines, while only a small proportion were related to the Johnson & Johnson’s vaccine (5.69%). The most common non-pregnancy specific adverse events were headache (482; 2.21%), fatigue (472; 2.16%), and pyrexia (436; 2.00%), while adverse pregnancy outcomes with the highest reporting rate were abortions spontaneous (762; 3.49%), and vaginal haemorrhage (229; 1.05%).

**Conclusion:**

This post-marketing survey on VAERS data have provided updated evidence on the safety of COVID-19 vaccines during pregnancy, thus supporting clinicians in recommending maternal immunization.

## Introduction

Most of trials on COVID-19 vaccines explicitly excluded pregnancy or failed to address pregnancy at all [1]. Without preauthorization human data on vaccines efficacy and safety during pregnancy, health care agencies’ statements on use in pregnant women can be issued only after favourable evidence is collected from the real-world clinical setting.

The U.S. Centers for Disease Control and Prevention (CDC) recommend COVID-19 vaccination for women who are pregnant, breastfeeding, trying to get pregnant now, or might become pregnant in the future [2]. As determined by CDC reviewers based on available literature about SARS-CoV-2 infection, pregnancy is listed as one of the underlying medical conditions that may increase the risk for severe illness from COVID-19 [3]. According to a recent meta-analysis on the impacts of SARS-CoV-2 infection during pregnancy, pregnant women infected with SARS-CoV-2 are at significantly increased risk of maternal mortality (RR 7.68; 95% CI 1.70 to 34.61), admission into an intensive care unit (3.81; 2.03 to 7.17), need for mechanical ventilation (15.23; 4.32 to 53.71) or any critical care (5.48; 2.57 to 11.72) as compared with uninfected pregnant women [4]. Moreover, pregnant women with COVID-19 are more likely to experience adverse pregnancy outcomes, such as preterm birth and stillbirth, as compared with pregnant women without COVID-19 [5]. Hence, vaccination against COVID-19 in pregnant women is crucial for preventing both maternal and infant morbidity and mortality.

As the number of pregnant women vaccinated against SARS-CoV-2 infection increases, post-marketing surveillance is essential for the ongoing evaluation of the safety of COVID-19 vaccines in pregnancy, and, if no issues emerge, also to provide confidence to pregnant women struggling with the choice of whether to get vaccinated or not. With the aim of providing further evidence on possible vaccine-related adverse reactions in pregnancy, we captured from the Vaccine Adverse Event Reporting System (VAERS) all adverse events (AEs) reported with COVID-19 vaccines occurred in pregnant women from December 2020 to April 2022.

## Methods

Data on adverse events following immunizations (AEFIs) with COVID-19 vaccines in pregnancy were retrieved from the open-access Vaccine Adverse Event Reporting System through the CDC’s WONDER System [6]. Since it was established in 1990, VAERS is part of the national monitoring system in place to capture possible side effects or health problems that might occur after any vaccination, including the newest ones against COVID-19. Reports in VAERS database are identified by a unique Case ID and include information on patient demographic characteristics, the type of vaccine administered, adverse events experienced, and their interval of onset. As per the U.S. Code of Federal Regulations, VAERS reports are further classified as serious if any of the following outcomes are reported: hospitalization or prolongation of hospitalization, permanent disability, life-threatening illness, death, or congenital anomaly [7]. Signs and symptoms of AEs entered VAERS database using the Medical Dictionary for Regulatory Activities (MedDRA), a standardized international medical terminology [8].

In order to collect reports of people who received a COVID-19 vaccine dose and who reported an AE to VAERS while pregnant, males and females over 50 years of age were excluded in the first place. To identify pregnancy-status, a query was then performed by searching for the term “Preg” in the symptom finder tool and selecting the following MedDRA codes: drug exposure during pregnancy, exposure during pregnancy, first trimester pregnancy, maternal exposure during pregnancy, multiple pregnancy, pregnancy, second trimester pregnancy, third trimester pregnancy, and twin pregnancy. Finally, we checked all reports one by one related to patients whose sex was not stated to ensure that the findings did not include neither foetal nor baby reports. The same assessment was also applied to reports concerning women whose age was unknown. When VAERS reports of pregnant women described events occurring both in the foetus or infant and in the mother, we considered all reported AEs and counted them separately.

For those reports that met our inclusion criteria and except for “Foreign” reports, we provided a descriptive analysis of the age distribution for pregnant women who experienced one or more AEs following COVID-19 vaccines (Table 1). Reported AEs were classified into non-pregnancy-specific (e.g., local and systemic reactions) and pregnancy-specific (e.g., spontaneous abortion, pre-eclampsia) adverse events [9], and then listed in Tables 2 and 3 respectively. For both Tables 2 and 3, events that were not evaluable as suspected adverse reactions (e.g., exposure during pregnancy, ultrasound scan, blood test) were excluded. Unfortunately, we could not breakdown the reports according to trimesters of pregnancy because most of them lacked the corresponding MedDRA code.

**Table 1:** Patients’ age and sex who expressed at least one adverse event related to COVID-19 vaccines

**Age**	**6–17 years**	**18–29 years**	**30–39 years**	**40–49 years**	**Unknown**	**Total**
N	39	1008	3029	301	492	4869
%	0.8	20.7	62.21	6.18	10.11	100

**Table 2:** Most reported non-pregnancy-specific adverse event in VAERS

**Non-pregnancy-specific Adverse Events**	**Events Reported**	**%**^a^
Headache	482	2.21
Fatigue	472	2.16
Pyrexia	436	2.00
Chills	396	1.82
Pain in extremity	383	1.76
Pain	344	1.58
Nausea	320	1.47
Dizziness	228	1.05
Myalgia	198	0.91
COVID-19	188	0.86
Vomiting	173	0.79
SARS-CoV-2 test positive	172	0.79
Injection site pain	150	0.69
Vaccination site pain	110	0.50
Dyspnoea	110	0.50
Arthralgia	109	0.50
Malaise	105	0.48
Muscle spasms	101	0.46
Rash	89	0.41
Haemorrhage	87	0.40
Abdominal pain	74	0.40
Injection site erythema	70	0.34
Diarrhoea	69	0.32
Hyperhidrosis	68	0.32
Pruritus	68	0.31
Chest pain	65	0.31
Cough	65	0.30
Palpitations	60	0.30
Back pain	57	0.28
Heart rate increased	56	0.26
Paraesthesia	55	0.26
Oropharyngeal pain	54	0.25
Hypoaesthesia	54	0.25
SARS-CoV-2 test negative	54	0.25
Ultrasound scan abnormal	54	0.25
Injection site pruritus	52	0.25
Urticaria	52	0.24

^a^The percentages refer to the total number of AEs reported in the VAERS database

**Table 3:** Most reported pregnancy-specific adverse event in VAERS

**Pregnancy-specific Adverse Events**	**Events Reported**	**%**^a^
Abortion spontaneous	762	3.49
Vaginal haemorrhage	229	1.05
Delivery	197	0.90
Ultrasound antenatal screen abnormal	150	0.69
Caesarean section	127	0.58
Foetal death	106	0.49
Foetal heart rate abnormal	94	0.43
Uterine dilation and curettage	92	0.42
Premature labour	89	0.41
Premature delivery	88	0.40
Ultrasound foetal abnormal	76	0.35
Induced labour	61	0.35
Pre-eclampsia	61	0.28
Stillbirth	56	0.28

^a^The percentages refer to the total number of AEs reported in the VAERS database

## Results

From December 2020 to April 1, 2022, a total of 4,869 reports involving pregnant women at COVID-19 vaccination were reported to VAERS. As shown in Table 1, most vaccine recipients belonged to the age group between 30 and 39 years old (3,029; 62.21%) and nearly half experienced an adverse event within 48 h of immunization (2,344; 48.14%), although some were observed up to 120 days and beyond.

Overall, 21,816 suspected adverse reactions associated with COVID-19 vaccines were reported, and they were described as serious as per the U.S. Code of Federal Regulations for 953 patients (19.57%), of whom 1/3 recovered [7]. Overall, nearly 80% of serious reports refer to hospitalizations, 22% to congenital anomalies or birth defects, 12% to life threatening outcomes, and 1% to deaths. Most of the vaccines reported, with a percentage of 53.34% and 40.68%, were related to mRNA-1273 and BNT162b2 respectively, while only a small proportion were related to the Johnson & Johnson’s vaccine (5.69%). With regard to mRNA-based COVID-19 vaccines, almost three quarters of serious reports occurred after immunization with the BNT162b2 vaccines, whereas 40% with the mRNA-1273 vaccine. Safety data on COVID-19 vaccination in pregnant women recovered from VAERS included both non-pregnancy-specific and pregnancy-specific adverse events [9]. The most common non-pregnancy specific outcomes were headache (482; 2.21%), fatigue (472; 2.16%), and pyrexia (436; 2.00%) (Table 2), while adverse pregnancy outcomes with the highest reporting rate were abortions spontaneous (762; 3.49%), and vaginal haemorrhage (229; 1.05%) (Table 3). Spontaneous abortions were mainly related to the BNT162b2 vaccine and occurred in about 16% of cases with an onset time of 0–1 days after immunization. Similarly, 75% of vaginal haemorrhages occurred following administration of the BNT162b2 vaccine and almost half within 7 days of vaccination.

## Discussion

The present survey provides an update of the COVID-19 vaccine safety profile in pregnancy. From the start of the U.S. vaccination program to April 1, 2022, VAERS received and processed 4,869 reports of AEs among pregnant women who received any of the available COVID-19 vaccines.

The non-pregnancy-specific adverse events most frequently reported in pregnant persons in VAERS were headache, fatigue, pyrexia, chills, pain in extremity, pain, nausea, and dizziness. Such pattern of events, which has already emerged in previous post-marketing safety studies [9–11], is largely consistent with the systemic reactogenicity profile of the Pfizer-BioNTech and the Moderna vaccines, which account for more than 90% COVID-19 vaccines administered to the pregnant population according to our survey. Based on the estimate rate of pregnant women exposed to COVID-19 vaccines provided through the V-safe surveillance system as of 4 April 2022 (215,713) [12], we found that the incidence rate for such events was between 1/1,000 and 1/100, i.e. uncommon. On the other hand, the frequency rates listed in the Summary of Product Characteristics (SPCs) are markedly higher for almost all of the above adverse reactions, which are ranked as very common (≥ 1/10) [13, 14]. Moreover, the participation in V-safe is voluntary, therefore the actual incidence is likely to be lower than determined. This result is consistent with previous observational studies evaluating adverse effects after maternal mRNA-based COVID-19 vaccination, which reported some systemic reactions, including headache, fever, chills, and myalgia, less frequently among pregnant than non-pregnant women [9, 15]. Nevertheless, the overall reactogenicity profiles seem to be similar and, according to the review by Badell et al., the available data do not support safety concerns with COVID-19 vaccines in pregnancy [16].

The adverse event following immunization with the highest reporting rate in VAERS was spontaneous abortion, i.e. a pregnancy-related event. Preliminary findings by Shimabukuro et al. [9] and the more recent ones by Moro et al. [10, 11] on COVID-19 vaccine safety in pregnancy from VAERS provided a similar result. Although the number of reports examined by Shimabukuro et al. was much lower, miscarriage already emerged as the most common adverse event among pregnant women during the first two months of U.S. vaccination program [9]. It accounted for 20.8% of all reports of AEs, whereas in our survey abortion amount to 15.7%. The unexpected loss of a pregnancy may happen by a variety of factors, the most important of which are mother-related. Among them, maternal age, prior obstetrical history, and maternal comorbidities are known to be important predictors in the risk of miscarriage [17]. As the information provided is often limited, maternal VAERS reports alone do not allow us to establish a link between spontaneous abortions and COVID-19 vaccination. Nonetheless, neither of the two large population-based case–control studies available to date found evidence of increased odds of early pregnancy loss associated with COVID-19 vaccine exposure in the previous three (adjusted odds ratio 0.91; 95% IC 0.75 to 1.10), four (1.02; 0.96 to 1.08) or five weeks (0.81; 0.69 to 0.95) [18, 19]. Furthermore, no increased risk of other adverse peripartum outcomes, such as postpartum haemorrhage, NICU admission, preterm birth, and stillbirth, has emerged from previous studies on COVID-19 vaccination in pregnancy, regardless of number of doses received during pregnancy or mRNA vaccine product [20, 21].The events reported to the VAERS database generally include not only suspected side effects, but also information on medical investigations or clinical characteristics, such as the pregnancy trimester. However, of all 4,869 reports involving pregnant women at COVID-19 vaccination, just 43 stated the stage of pregnancy (23 referred to the first trimester, 11 to the second, and 9 to the third).

Some important limitations of the present COVID-19 vaccine safety survey should be noted. First, given the lack of information on the trimester of exposure in most reports, we were not able to relate adverse events according to the stage of pregnancy during which the patients underwent vaccination. Since VAERS is a passive surveillance system, anyone can submit a report on their experience after vaccination, including general public; this can lead to mistakes in filling in the report and so to a low quality of the information provided. In addition, attempts by VAERS scientists to contact the sender often fail, preventing up-to-date monitoring of adverse events during the pregnancy. Under-reporting, on the other hand, which is the main limitation VAERS is subject to, is expected to be very low because the data were collected during the most intensive vaccine safety monitoring effort in U.S. history, and especially because reporting of serious adverse events in pregnancy by health professionals is required by the CDC.

## Conclusion

As more pregnant women get vaccinated against COVID-19, ongoing safety monitoring is crucial for the evaluation of the real safety profile in this vulnerable population. This post-marketing survey on VAERS data provided updated evidence on the safety of COVID-19 vaccines during pregnancy, supporting clinicians in recommending maternal immunization.

## Availability of data

The data that support the findings of this study are available from the corresponding author upon reasonable request.
